# Speed/Accuracy Trade-Off between the Habitual and the Goal-Directed Processes

**DOI:** 10.1371/journal.pcbi.1002055

**Published:** 2011-05-26

**Authors:** Mehdi Keramati, Amir Dezfouli, Payam Piray

**Affiliations:** 1 School of Management and Economics, Sharif University of Technology, Tehran, Iran; 2 Control and Intelligent Processing Center Of Excellence, School of Electrical and Computer Engineering, University of Tehran, Tehran, Iran; University of Oxford, United Kingdom

## Abstract

Instrumental responses are hypothesized to be of two kinds: habitual and goal-directed, mediated by the sensorimotor and the associative cortico-basal ganglia circuits, respectively. The existence of the two heterogeneous associative learning mechanisms can be hypothesized to arise from the comparative advantages that they have at different stages of learning. In this paper, we assume that the goal-directed system is behaviourally flexible, but slow in choice selection. The habitual system, in contrast, is fast in responding, but inflexible in adapting its behavioural strategy to new conditions. Based on these assumptions and using the computational theory of reinforcement learning, we propose a normative model for arbitration between the two processes that makes an approximately optimal balance between search-time and accuracy in decision making. Behaviourally, the model can explain experimental evidence on behavioural sensitivity to outcome at the early stages of learning, but insensitivity at the later stages. It also explains that when two choices with equal incentive values are available concurrently, the behaviour remains outcome-sensitive, even after extensive training. Moreover, the model can explain choice reaction time variations during the course of learning, as well as the experimental observation that as the number of choices increases, the reaction time also increases. Neurobiologically, by assuming that phasic and tonic activities of midbrain dopamine neurons carry the reward prediction error and the average reward signals used by the model, respectively, the model predicts that whereas phasic dopamine indirectly affects behaviour through reinforcing stimulus-response associations, tonic dopamine can directly affect behaviour through manipulating the competition between the habitual and the goal-directed systems and thus, affect reaction time.

## Author Summary

When confronted with different alternatives, animals can respond either based on their pre-established habits, or by considering the short- and long-term consequences of each option. Whereas habitual decision making is fast, goal-directed thinking is a time-consuming task. Instead, habits are inflexible after being consolidated, but goal-directed decision making can rapidly adapt the animal's strategy after a change in environmental conditions. Based on these features of the two decision making systems, we suggest a computational model using the reinforcement learning framework, that makes a balance between the speed of decision making and behavioural flexibility. The behaviour of the model is consistent with the observation that at the early stages of learning, animals behave in a goal-directed way (flexible, but slow), but after extensive learning, their responses become habitual (inflexible, but fast). Moreover, the model explains that the animal's reaction time must decrease through the course of learning, as the habitual system takes control over behaviour. The model also attributes a functional role to the tonic activity of dopamine neurons in balancing the competition between the habitual and the goal-directed systems.

## Introduction

A very basic assumption in theories of animal decision making is that animals possess a complicated learning machinery that aims for maximizing rewards and minimizing threats to homeostasis [1]. The primary question within this framework is then how the brain, constrained by computational limitations, uses past experiences to predict rewarding and punishing consequences of possible responses.

The dual-process theory of decision making proposes that two distinct brain mechanisms are involved in instrumental responding: the “habitual”, and the “goal-directed” systems [2]. The habitual system is behaviourally defined as being insensitive to outcome-devaluation, as well as contingency-degradation. For example, in the experimental paradigm of outcome-devaluation, the animal is first trained for an extensive period to perform a sequence of actions for gaining access to a particular outcome. The outcome is then devaluated by being paired with an aversive stimuli (conditioned taste-aversion), or by over-consumption of that outcome (sensory-specific satiety). The critical observation is that in the test phase, which is performed in extinction, the animal continues responding for the outcome, even though it is devaluated. The goal-directed process, on the other hand, is defined as being sensitive to outcome-devaluation and contingency-degradation. This behavioural sensitivity is shown to emerge when the pre-devaluation training phase is limited, rather than extensive Adams [3].

Based on these behavioural patterns, two different types of associative memory structures are proposed for the two systems. The behavioural autonomy demonstrated by the habitual system is hypothesized to be based on the establishment of associations between contextual stimuli and responses (S-R), whereas representational flexibility of the goal-directed system is suggested to rely on associations between actions and outcomes (A-O).

A wide range of electrophysiological, brain imaging, and lesion studies indicate that different, and topographically segregated cortico-striato-pallido-thalamo-cortical loops underlie the two learning mechanisms discussed above (see [4] for review). The sensorimotor loop, comprising of glutamatergic projections from infralimbic cortices to dorsolateral striatum, is shown to be involved in habitual responding. In addition, phasic activity of dopamine (DA) neurons, originating from midbrain and projecting to different areas of the striatum is hypothesized to carry a reinforcement signal, that is shown to play an essential role in the formation of S-R associations. The associative loop, on the other hand, is proposed to underlie goal-directed responding. Some critical components of this loop include dorsomedial striatum and paralimbic cortex.

The existence of two parallel neuronal circuits involved in decision making arises the question of how the two systems compete for taking control over behaviour. Daw and colleagues, proposed a reinforcement learning model in which, the competition between the two systems is based on the relative uncertainty of the systems in estimating the value of different actions [5]. Their model can explain some behavioural aspects of interaction between the two systems. A critical analysis of their model is provided in the discussion section.

In this paper, based on the model proposed in [5], and using the idea that reward maximization is the performance measure of the decision making system of animals, we propose a novel, normative arbitration mechanism between the two systems that can explain a wider range of behavioural data. The basic assumption of the model is that the habitual system is fast in responding, but inflexible in adapting its behavioural strategy to new conditions. The goal-directed system, in contrast, can rapidly adapt its instrumental knowledge, but is considerably slower than the habitual system in making decisions. In the proposed model, not only the two systems seek to maximize the accrual of reward -by different algorithms-, but the arbitration mechanism between them is also designed in a way to exploit the comparative advantages of the two systems in value estimation.

As a direct experimental observation for supporting the assumptions of the model, it has been reported classically that when rats traverse a T-maze to obtain access to an outcome, at the choice points, they pause and vicariously sample the alternative choices before committing to a decision [6]–[8]. This behaviour, called “vicarious trial-and-error” (VTE), is defined by head movements from one stimulus to another at a choice point, during simultaneous discrimination learning [9]. This hesitation- and conflict-like behaviour is suggested to be indicative of deliberation or active processing by a planning system [6], [7], [10], [11]. Important for our discussion, it has been shown that after extensive learning, VTE frequency declines significantly [6], [12], [13]. This observation is interpreted as a transition of behavioural control from the planning system to the habitual one, and shows difference in the decision-time between habitual and goal-directed responding [14].

Beside being supported by the VTE behaviour, the assumption about the relative speed and flexibility of the two systems allows the model to explain some behavioural data on choice reaction time. The model also predicts that whereas phasic activity of DA neurons indirectly affects the arbitration through intervening in habit formation, tonic activity of DA neurons can directly influence the competition by modulating the cost of goal-directed deliberation.

## Model

### The Preliminaries

Reinforcement learning (RL) is learning how to establish different types if instrumental associations for the purpose of maximizing the accrual of rewards [15]. In the RL framework, stimuli and responses are referred to as states and actions, respectively. An RL agent perceives its surrounding environment in the form of a finite set of states, 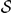, in each of which, one action among a finite set of actions, 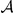, can be taken. The dynamics of the environment can be formulated by a transition function and a reward function. The transition function, denoted by 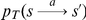, represents the probability of reaching state 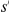 after taking action 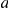 at state 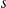. The reward function, 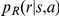, indicates the probability of receiving reward 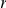, by executing action 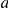 at state 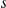. This structure, known as the Markov Decision Process (MDP), can be demonstrated by a 4-tuple, 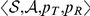. At each time-step, 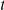, the agent is in a certain state, say 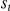, and makes a choice, say 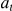, from several alternatives on the basis of subjective values that it has assigned to them through its past experiences in the environment. This value, denoted by 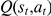, is aimed to be proportional to the sum of discounted rewards that are expected to be received after taking action 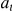 onward:



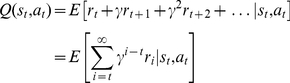
(1)



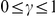
 is the discount factor, which indicates the relative incentive value of delayed rewards compared to immediate ones.

Model-free and model-based RL, are two variants of reinforcement learning with behavioural characteristics similar to the habitual and goal-directed systems, respectively [5]. These two variants are in fact two different mechanisms for estimating the 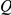-function of equation 1 , based on the feedbacks, 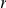, that the animal receives from the environment through learning.

In temporal difference RL (TDRL), which is an implementation of model-free RL, a prediction error signal, 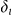, is calculated each time the agent takes an action and receives a reward from the environment. This prediction error is calculated by comparing the prior expected value of taking that action, 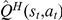, with its realized value after receiving reward, 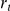:



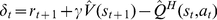
(2)



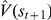
 is the maximum value of all feasible actions available at 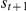. The prediction error signal is hypothesized to be carried by the burst firing of midbrain dopamine neurons. This signal can be used to update the estimated value of actions:



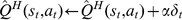
(3)



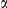
 is the learning rate, representing the degree to which the prediction error adjusts the 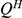-values of the habitual system. Assuming that the reward and transition functions of the environment are stationary, equations 2 and 3 will lead the 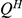-values to eventually converge through learning to the expected sum of discounted rewards. Therefore, after a sufficiently long learning period, the habitual system will be equipped with the instrumental knowledge required for taking the optimal behavioural strategy. This optimal decision making is achievable without the agent knowing the dynamics of the environment. This is why this mechanism is known as model-free reinforcement learning. The gradual convergence of 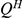-values to their steady levels, leads the habitual system toward being insensitive to sudden changes in the environment's dynamics, such as outcome-devaluation and contingency degradation. Instead, as all the information required for making a choice between several alternatives is cached in S-R associations through the course of learning, the habitual responses can be made within a short interval after the stimulus is presented.

Instead of keeping and updating point estimations, by using Kalman reinforcement learning [16], the habitual system in our model keeps probability distributions for the 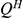-values of each state-action pair (See Methods for mathematical details). These probability distributions contain substantial information that will be later used for arbitration between the habitual and the goal-directed systems.

In contrast to the habitual process, the value estimation mechanism in a model-based RL is based on the transition and reward functions that the agent has learned through past experiences [5], [15]. In fact, through the course of learning, the animal is hypothesized to learn the causal relationship between various actions and their outcomes, as well as the incentive value of different outcomes. Based on the former component of the environment's dynamics, the goal-directed system can deliberate the short-term and long-term consequences of each sequence of actions. Then by using the learned reward function, calculating the expected value for each action sequence will be possible.

Letting 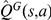 denote the value of each action calculated by this method, the recursive value-iteration algorithm below can compute it (See Methods for algorithmic details):



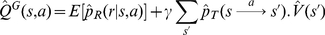
(4)


Due to employing the estimated model of the environment for value estimation, the goal-directed system can rapidly revise the estimated values after an environmental change, as soon as the transition and reward functions are adapted to the new conditions. This can explain why the goal-directed system is sensitive to outcome-devaluation and contingency-degradation [5]. But according to this computational mechanism, one would expect the value estimation by the goal-directed system to take a considerable amount of time, as compared to the habit-based decision time. The difference in speed and accuracy of value estimation by the habitual and goal-directed processes is the core assumption of the arbitration mechanism proposed in this paper, that allows the model to explain a set of behavioural and neurobiological data.

### Speed/Accuracy Trade-off

If we assume for simplicity that the goal-directed system is always perfectly aware of the environment's dynamics, then it can be concluded that this system has perfect information about the value of different choices at each state. This is a valid assumption in most of the experimental paradigms considered in this paper. For example, in outcome-devaluation experiments, due to the existence of a re-exposure phase between training and test phases, the subjects have the opportunity to learn new incentive values for the outcomes. Although the goal-directed system, due to its flexible nature, will always have “more accurate” value estimations compared to the habitual system, the assumption of having “perfect” information might be violated under some conditions (like reversal learning tasks). This violation will naturally lead to some irrational arbitrations between the systems.

Thus, the advantage of using the goad-directed system can be approximated by the advantage of having perfect information about the value of actions. But this perfect information can be extracted from transition and reward functions at the cost of losing time; a time which could be instead used for taking rapid habitual actions and thus, receiving less rewards in magnitude, but more in frequency. This trade-off is the essence of the arbitration rule between the two systems that we propose here. In other words, we hypothesize that animals balance the benefits of deliberations against their cost. Its benefit is proportional to the value of having perfect information, and its cost is equal to the potential reward that could be acquired during the time that the organism is waiting for the goal-directed system to deliberate.

**Figure 1:**
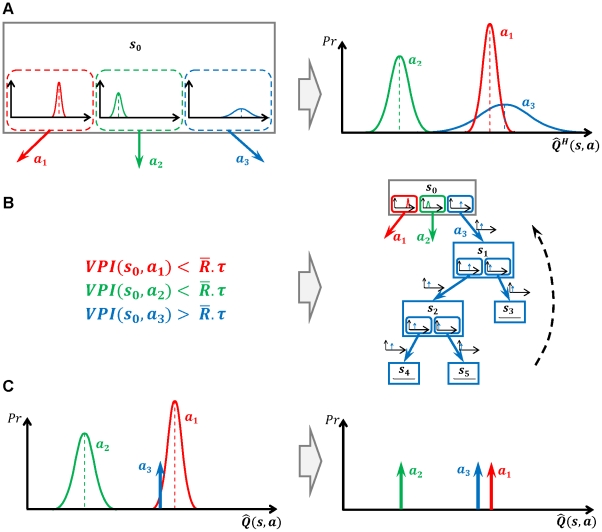
An example for showing the proposed arbitration mechanism between the two processes. (A) The agent is at state 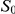 and three choices are available: 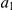, 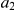 and 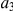. The habitual system, as shown, has an estimate for the value of each action in the form of probability distribution functions, based on its previous experiences. These uncertain estimated values are then compared to each other in order to calculate the expected gain of having the exact value of each action (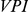). In the case of this example, action 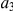 has the highest mean value, according to the uncertain knowledge in the habitual system. However, it is probable that the exact value of this action be less than the mean value of action 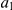. In that case, the best strategy would be to choose action 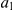, rather that 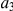. Thus, it is worth knowing the exact value of 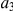 (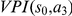 has a high value). (B) The exact value of actions is supposed to be attainable if a tree search is performed in the decision tree, by the goal-directed system. However, the benefit of search must be higher than its cost. The benefit of deliberation for each action is equal to its 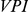 signal, whereas the cost of deliberation is equal to 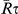, which is the total reward that could be potentially acquired during the deliberation time, 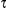 (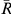 is the average over acquired rewards during some past actions). Since for action 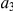, the benefit of deliberation has exceeded its cost, the goal-directed system is engaged in value estimation. (C) Finally, action selection is carried out based on the estimated values for actions, which have come from either the habitual (for actions 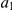 and 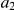) or the goal-directed (for action 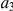) system. For those actions that are not deliberated, the mean value of their distribution function is used for action selection.

As illustrated schematically in Figure 1 , at each time-step, the habitual system has an imperfect estimate for the value of each action in the form of a distribution function. Using these distribution functions, the expected benefit of estimating the value of each action 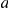 by the goal-directed system is computed (see below). This benefit, called “value of perfect information”, can be denoted by 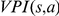. The cost of deliberation, denoted by 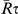, is also computed separately (See below). Having the cost and benefit of deliberation for each action, if the benefit is greater than the cost, i.e. 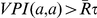, the arbitrator will decide to run the goal-directed system for estimating the value of action 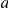; otherwise, the value of action 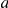 that will be used for action selection will be equal to the mean of the distribution function cached in the habitual system for that action. Finally, based on the estimated values of different actions that have been derived from either of the two instrumental systems, a softmax action selection rule, in which the probability of choosing each action increases exponentially with its estimated value, can be used (See Methods). Upon executing the selected action and consequently receiving a reward and entering a new state, both the habitual and goal-directed systems will update their instrumental knowledge for future exploitations.

Based on the decision theoretic ideas of “value of information” [17], a measure has been proposed in [18] for information value in the form of expected gains in performance, resulted from improved policies if perfect information was available. This measure, which is computed from probability distributions over the 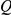-value of choices, is used in the original paper for proposing an optimal solution for the exploration/exploitation trade-off. Here, we use the same measure for estimating the benefit of goal-directed search.

To see how this measure can be computed, assume that the animal is in the state 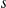, and one of the available actions is 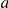, with the estimated value 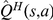 assigned to it by the habitual system. At this stage, we are interested to know how much the animal will benefit if it understands that the true value of actions 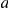 is equal to 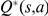, rather than 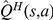. Obviously, any new information about the exact value of an action is valuable only if it improves the previous policy of the animal that was based on 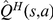. This can happen in two scenarios: (a) when knowing the exact value signifies that an action previously considered to be sub-optimal is revealed to be the best choice, and (b) when the new knowledge shows that the action which was considered to be the best, is actually inferior to some other actions. Therefore, the gain of knowing that the true value of 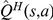 is 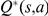 can be defined as [18]:



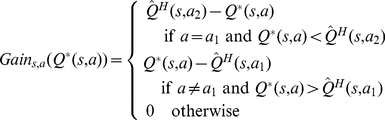
(5)



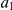
 and 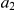 are the actions with the best and second best expected values, respectively. In the definition of the gain function, the first and the second rules correspond to the second and the first scenarios discussed above, respectively.

According to this definition, calculating the gain function for each choice requires knowing the true value of that state-action pair, 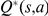, which is unavailable. But, as the habitual system is assumed to keep a probability distribution function for the value of actions, the agent has access to the probability of possible values of 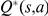. Using this probability distribution of 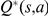, the animal can take expectation over the gain function to estimate the value of perfect information (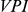):



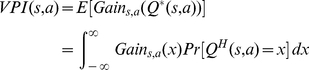
(6)


Intuitively, and crudely speaking, the value of perfect information for an action is somehow proportional to the overlap between the distribution function of that action and the distribution function of the expectedly best action. Exceptionally, for the case of the expectedly best action, the 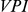 signal is proportional to the overlap between its distribution function and the distribution function of the expectedly second best action. It is worth to emphasize that for the calculation of 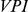 signals, the goal-directed system has in no way been involved and instead, all the necessary information has been provided by the habitual process. The 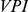 signal for an action expresses the degree to which having perfect information about that action, i.e. knowing its true value, results in policy improvement and thus, 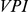 is indicative of the benefit of deliberation.

It is worth mentioning that computing the 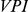 integral proposed in equation 6 is shown to have a closed form equation [18] and thus, the integral doesn't need to be actually taken. Therefore, assuming that the time needed for evaluating 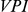 is considerably less than that of running the goal-directed system is plausible.

For computing the cost of deliberation, on the other hand, assuming that deliberation about the value of each action takes a fixed time, 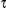, the cost of deliberation can be quantified as 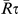; where 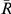 is the average rate of reward per time unit. Average reward can be interpreted as the opportunity cost of latency in responding to the environmental stimuli [19]. It means that when the average reward has a high value, every second in which a reward is not obtained is costly. Average reward can be computed as an exponentially-weighted moving average of obtained rewards:



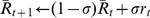
(7)


The arbitration mechanism proposed above, is an approximately optimal trade-off between speed and accuracy of responding. This means that given that the assumptions are true, the arbitration mechanism calls or doesn't call the goal-directed system, based on the criterion that sum of discounted rewards, as defined in equation 1 , should be maximized [See Methods for optimality proof]. The most challenging assumption, as mentioned before, is that the goal-directed system is assumed to have perfect information on the value of choices. As some cases that challenge the validity of this assumption one could mention the cases where only the goal-directed system is affected (for example after receiving some verbal instructions by the subject). Clearly, the cached values in the habitual system and thus the 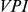 signal will not be affected under such treatments, though the real accuracy that the goal-directed system has in estimating values has changed.

## Results

### Outcome-Sensitivity after Moderate vs. Extensive Training

First discovered by Adams [3] and later replicated in a lengthy series of studies [20]–[23], it has been shown that the effect that the devaluation of outcome exerts on the animal's responses depends upon the extent of pre-devaluation training; i.e. responses are sensitive to outcome devaluation after moderate training, whereas overtraining makes responding insensitive to devaluation.

**Figure 2:**
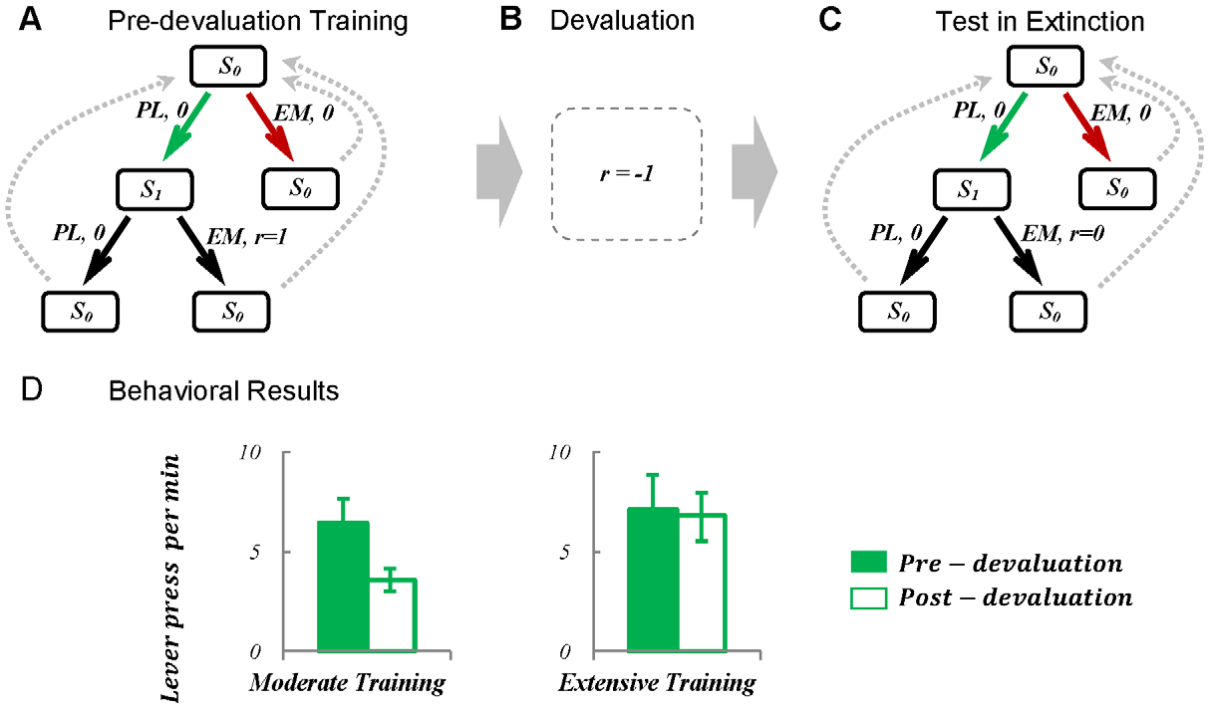
Formal representation of the devaluation experiment with one lever and one outcome, and behavioural results. (A) In the training phase, the animal is put in a Skinner box where pressing the lever 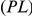 followed by a nose-poke entry in the food magazine (enter-magazine: 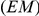) leads to obtaining the food reward. Other action sequences, like entering the magazine before pressing the lever (i.e. 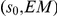) result in no reward. As the task is supposed to be cyclic, the agent will return back to the initial state, 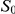, after taking each sequence of responses. (B) In the second phase, the devaluation phase, the food outcome which used to be acquired during the training period is devalued by being paired with illness. (C) The animal's behaviour is then tested in the same Skinner box used for training, with the difference that no outcome is delivered to the animal anymore, in order to avoid changes in behaviour due to new reinforcement. (D) Behavioural results (adopted from ref [22]) show that the rate of pressing the lever decreases significantly after devaluation for the case of moderate pre-devaluation training. In contrast, it doesn't show a significant change, when the training period has been extensive. Error bars represent 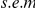 (standard error of the mean).

To check the validity of the proposed model, the model has been simulated in a schedule analogous to those used in the above mentioned experiments. The formal representation of the task, which was first suggested in [5], is illustrated in Figure 2 . As the figure shows, the procedure is composed of 3 phases. The agent is first placed in an environment where pressing the lever (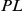) followed by entering the food magazine (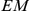) results in obtaining a reward with the magnitude of one; but magazine entry before lever press, or pressing the lever and not entering the magazine leads to no reward. As the task is supposed to be cyclic, after performing each chain of actions, the agent goes to the initial state and will start afresh (Figure 2:A). After a certain amount of training in this phase, the food outcome is devalued by being paired with poison, which is aversive with magnitude of one (equivalently, its reward is equal to -1) (Figure 2:B). Finally, to assess the effect of devaluation, the performance of the agent is measured in extinction, i.e. in the absence of any outcome (neither appetitive, nor aversive), in order to avoid the instrumental associations acquired during training from being affected in the test phase (Figure 2:C).

The behavioural results, as illustrated in Figure2:D, show that behavioural sensitivity to goal-devaluation depends on the extent of pre-devaluation training. In the moderate training case, the rate of responding has significantly decreased after devaluation, which is an indicator of goal-directed responding. However, after extensive training, no significant sensitivity to devaluation of the outcome is observed, implying that responding has become habitual.

**Figure 3:**
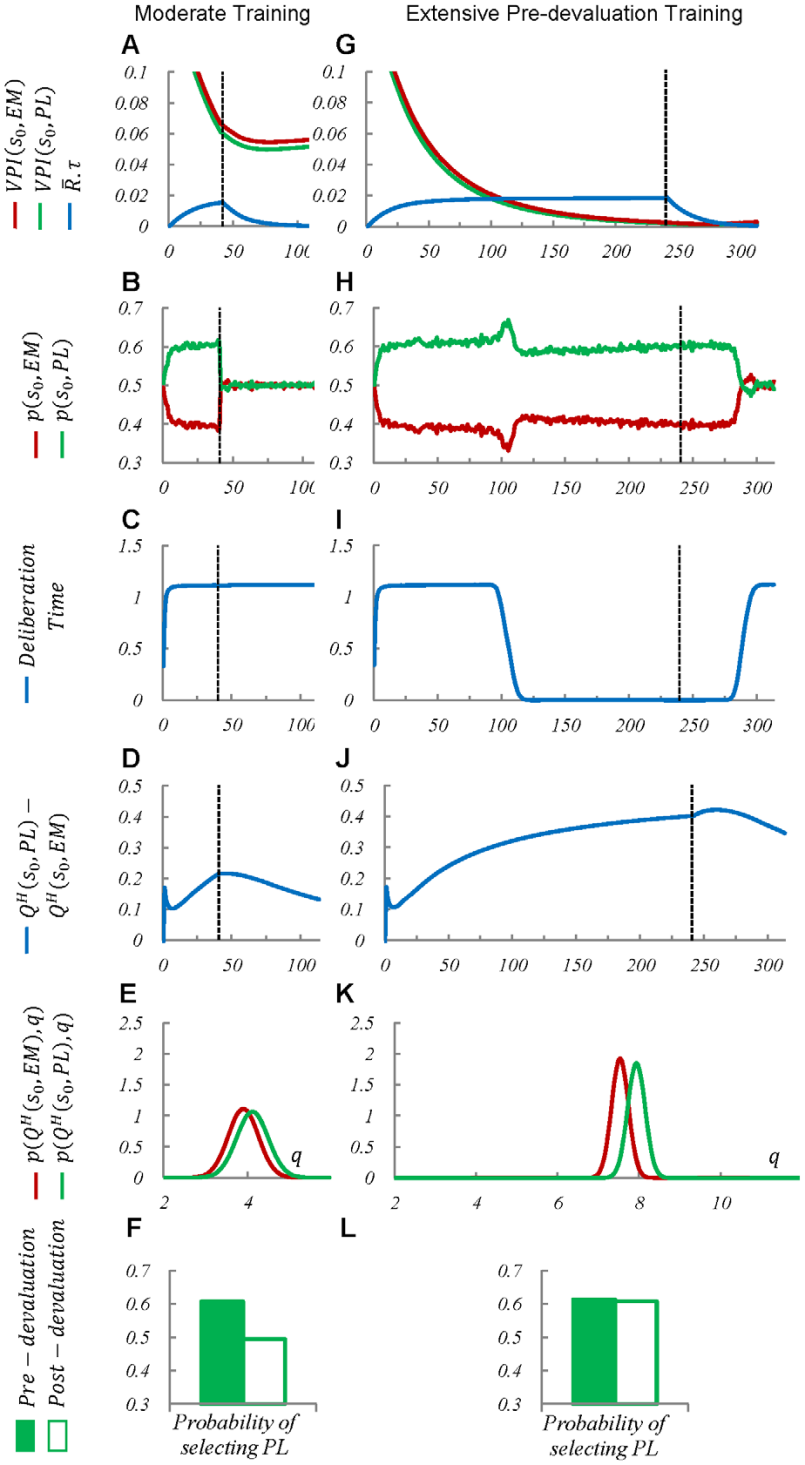
Simulation results of the model in the schedule depicted in **Figure 2**. The model is simulated under two scenarios: moderate training (left column), and extensive training (right column). In the moderate training scenario, the agent has experienced the environment for 40 trials before devaluation treatment, whereas in the extensive training scenario, 240 pre-devaluation training trials have been provided. In sum, the figure shows that after extensive training, but not moderate training, the 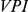 signal is below 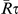 at the time of devaluation (Plot 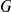 against 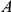). Thus, the behaviour in the second scenario, but not the first, doesn't change right after devaluation (Plot 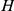 against 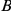. Also, plot 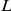 against 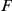). The low value of the 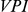 signal at the time of devaluation for the second scenario is because there is little overlap between the distribution functions of the values of the two available choices (Plots 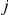 and 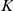). The opposite is true for the first scenario (Plots 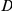 and 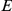). Numbers along the horizontal axis in plots 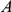 to 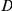, and 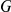 to 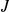, represent trial numbers. Each “trial” ends when the simulated agent receives a reward; e.g. in the schedule of Figure 2 , each time the agent chooses 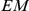 at state 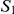, the trial number is counted up. Plots 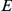 and 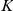 show the distribution functions of the habitual system over its estimated 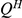-values, at one trial before devaluation. Bar charts 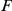 and 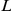 show the average probability of performing 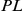 at 10 trials before (filled bars) and 10 trials after (empty bars) devaluation. All data reported are means over 3000 runs. The 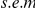 for all bar charts is close to zero and thus, not illustrated.

Through numerical simulation, homogeneous agents, i.e. agents with equal free parameters of the model, have carried out the experimental procedure under two scenarios: moderate vs. extensive pre-devaluation training. The only difference between the two scenarios is in the number of training trials in the first phase of the schedule: 40 trials for the moderate, and 240 trials for the extensive training scenario. The results are illustrated separately for these two scenarios in Figure 3 . It must be noted that since neither the “lever-press” nor the “enter-magazine” actions are performed by the animal during the devaluation phase, the habitual knowledge remains intact in this period; i.e. the habitual system is not simulated during the devaluation period. Devaluation is assumed to only affect the reward function, used by the goal-directed system.

Figure 3:A and G show that at the early stages of learning, the 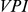 signal has a high value for both of the actions, 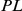 and 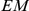, at the initial state, 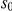. This indicates that due to initial ignorance of the habitual system, knowing the exact value of both of the actions will greatly improve the agent's behavioural strategy. Hence, the benefit of deliberation is more than its cost, 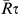. By obtaining a reward, the 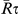 signal elevates gradually. Concurrently, as the 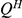-values estimated by the habitual process for the two actions converge to their real values through learning, the difference between them increases (Figure 3:D and J). This increase leads to the overlap between the distribution functions over the two actions becoming less and less (Figure 3:E and K) and consequently, the 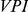 signal decreasing gradually.

Now by focusing on the moderate training scenario, it is clear that when devaluation has occurred at the trial number 40, the 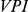 signals have not yet become less than 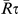 (Figure 3:A). Thus, the actions have been goal-directed at the time of devaluation and hence, the agent's responses have shown a great sensitivity to devaluation at the very early stages after devaluation; i.e. the probability of choosing action 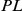 has sharply decreased to 50%, which is equal to that of action 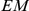 (Figure 3:B and F). Figure 3:C also shows that in the moderate training scenario, deliberation time has always been high; indicating that actions have always been deliberated using the goal-directed system.

In contrast to the moderate training scenario, the 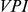 signal is below 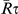 at the time of devaluation in the extensive training scenario (Figure 3:G). This means that at this point of time, the cost of devaluation has exceeded its benefit and hence, actions are chosen habitually. This can be seen in Figure 3:I, where deliberation time has reached zero after almost 100 training trials. As a consequence, the agent's responses have not sharply changed after devaluation (Figure 3:H and L). Because the test has been performed in extinction, the average reward signal has gradually decreased to zero after devaluation and concurrently, the 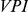 signal has slowly raised again, due to the reduction of the difference between the 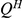-values of the two choices (Figure 3:J) and so, the augmentation of the overlap between their distribution functions. At the point that 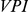 has exceeded 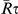, the agent's responses have become goal-directed again and so, deliberation time has boosted (Figure 3:I). Consistently, the rate of selection of each of the two choices has been adapted to the post-devaluation conditions (Figure 3:H).

In a nutshell, the simulation of the model in these two scenarios is consistent with the behavioural observation that moderately trained behaviours are sensitive to outcome devaluation, but extensively trained behaviours are not. Moreover, the model predicts that after extensive training, deliberation time declines; a prediction that is consistent with the VTE behaviour observed in rats [6]. Furthermore, the model predicts that deliberation time increases with a lag after devaluation in the extensive training scenario, whereas it remains unchanged before and after devaluation in the moderate training scenario.

Just for the sake of more clarification, the reason that the mean value of 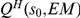 in Figures 3:E and K is above zero is because of the cyclic nature of the task, i.e. by taking action 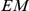 at state 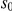, the agent goes back to the same state, which might have a positive value.

### Outcome-Sensitivity in a Concurrent Schedule

**Figure 4:**
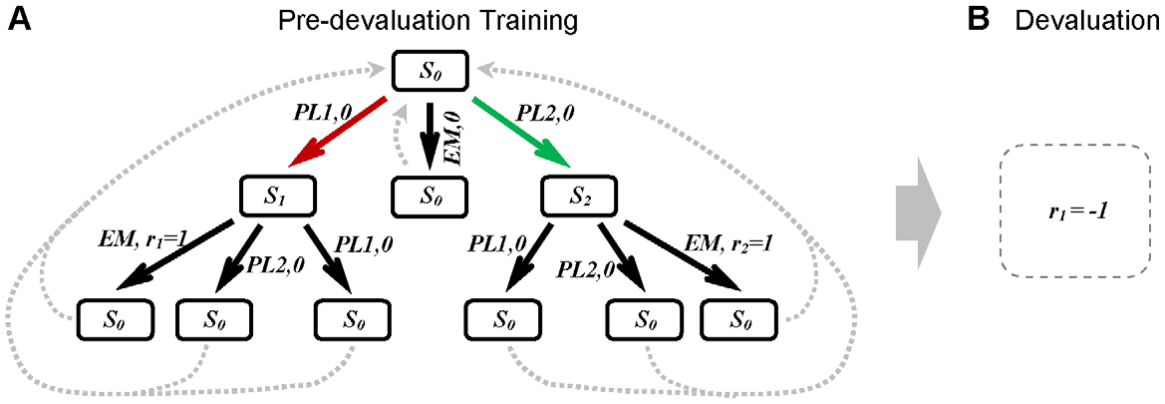
Tree representation of the devaluation experiment with two levers available concurrently. (A) In the training phase, either pressing lever one 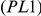 or pressing lever two 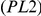, if followed by entering the magazine 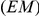, results in acquiring one unit of either of the two rewards, 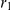 or 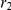, respectively. The reinforcing value of the two rewards is equal to one. Other action sequences lead to no reward. As in the task of Figure 2 , this task is also assumed to be cyclic. (B) In the devaluation phase, the outcome of one of the responses (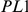) is devalued (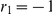), whereas the rewarding value of the outcome of the other response (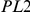) has remained unchanged. After the devaluation phase, the animal's behaviour is tested in extinction (for space consideration, this phase is not illustrated). Similar to the task of Figure 2 , neither 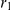 nor 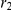 is delivered to the animal in the test phase.

The focus of the previous section was on simple tasks with only one response for each outcome. In another class of experiments, the development of behavioural autonomy has been assessed in more complex tasks where two different responses produce two different outcomes [21], [24]–[26]. Among those experiments, to the best of our knowledge, it is only in the experiment in [26] that the two different choices (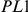 and 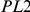) are concurrently available and hence, the animal is given a choice between the two responses (Figure 4:A). In the others, the two different responses are trained and also tested in separate sessions and so, their schedules are not compatible with the requirements of the reinforcement learning framework that is used in our model.

In [26], rats received extensive concurrent instrumental training in a task where pressing the two different levers produces different types of outcomes: food pellets and sucrose solution. Although the outcomes are different, they have equal reinforcing strength, in terms of the response rates supported by them. A task similar to that used in their experiment is formally depicted in Figure 4.

After extensively reinforcing the two responses, one of the outcomes was devalued by flavour aversion conditioning, as illustrated in Figure 4:B. Subsequently, given a choice between the two responses, the sensitivity of instrumental performance to this devaluation was assessed in extinction tests. The results of their experiment showed that devaluation reduced the relative performance of the response associated with the devalued outcome at the very early stage of the test phase, even after extensive training. Thus, it can be concluded that whatever the amount of instrumental training, S-R habits do not overcome goal-directed decision making when two responses with equal affective values are concurrently available.

**Figure 5:**
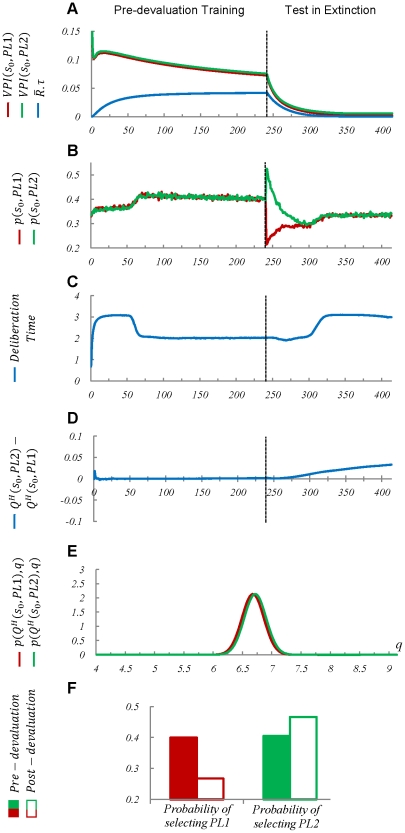
Simulation results for the task of **Figure 4**. The results show that since the reinforcing value of the two outcomes is equal, there is a huge overlap between the distribution functions over the 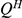-values of actions 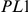 and 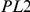, at state 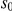, even after extensive training (240 trials) (Plots 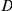 and 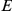). Accordingly, the 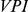 signals (benefit of goal-directed deliberation) for these two actions remain higher than the 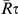 signal (cost of deliberation) (Plot 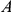) and thus, the goal-directed system is always engaged in value-estimation for these two choices. The behaviourally observable result is that responding remains sensitive to revaluation of outcomes, even though devaluation has happened after a prolonged training period (Plots 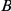 and 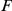).

Simulating the proposed model in the task of Figure 4 has replicated this behavioural observation. As illustrated in Figure 5:A, initially, the 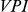 signal for the two responses has a high value which gradually decreases over time as the variance of the distribution functions over the estimated values of the two responses decreases; meaning that the habitual process becomes more and more certain about the estimated values. However, due to the forgetting effect, i.e. the habitual system forgets very old samples and does not use them in approximating the distribution function, the variance of the distribution functions over the values of actions doesn't converge to zero, but instead, converges to a level higher than zero. Moreover, because the strength of the two reinforcers is equal, as revealed in Figure 5:D ,the distribution functions do not get divorced (Figure 5:E). As a result of these two facts, the 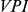 signal has converged at a level higher than 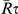 (Figure 5:A). This has led to the performance remaining goal-directed (Figure 5:C) and sensitive to devaluation of one of the outcomes; i.e. after devaluing the outcome of the action 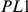, its rate of selection has sharply decreased and instead, the probability of selecting 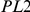 has increased (Figure 5:B and F).

As it is clear from the above discussion, the relative strength of the reinforcers critically affects the arbitration mechanism in our model. In fact, the model predicts that when the affective values of the two outcomes are close enough to each other, the 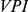 signal will not decline and hence, the behaviour will remain goal-directed and sensitive to devaluation, even after extensive training. But if the two outcomes have different reinforcing strength, then their corresponding distribution functions will gradually get divorced and thus, the 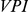 signal will converge to zero. This leads to the habitual process taking control of behaviour and the performance becoming insensitive to outcome devaluation. This prediction is in contrast to the model proposed in [5], in which the arbitration between the two systems is independent of the relative incentive values of the two outcomes. In fact, in that model, whether the value of an action comes from the habitual or the goal-directed system, only depends on the uncertainty of the two systems about their estimated values and thus, the arbitration between the two systems is independent of the estimated value for other actions.

### Reaction-Time in a Reversal Learning Task

**Figure 6:**
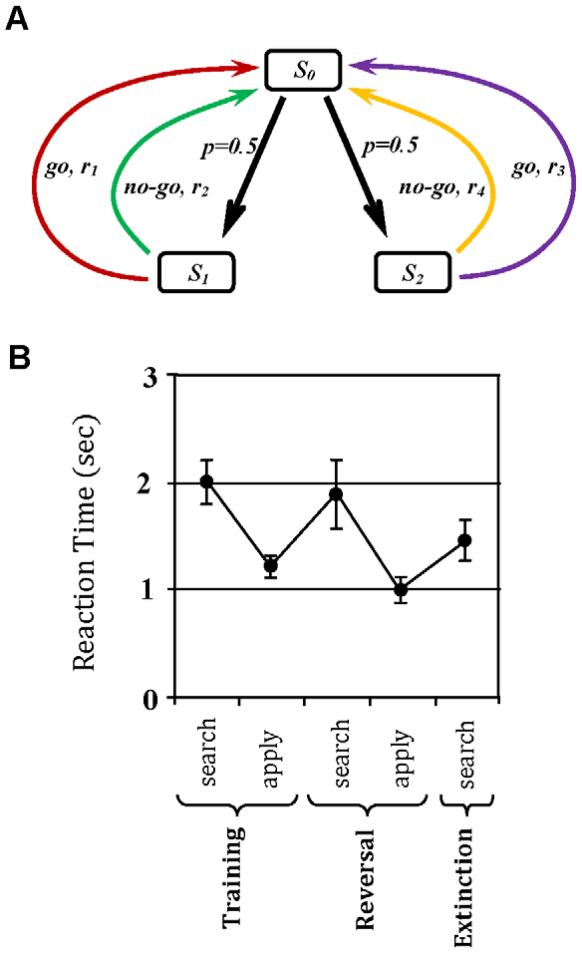
Tree representation of the reversal learning task, used in [**27**], and the behavioural results. (A) When each trial begins, one of the two stimuli, 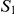 or 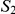, is presented in random on a screen. The subject can then choose whether to touch the screen (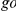 action) or not (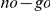 action). The task is performed in three phases: training, reversal, and extinction. During the training phase, the subject will receive a reward if the stimulus 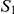 is presented and the action 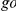 is performed by the subject, or if the stimulus 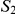 is presented and the action 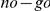 is selected (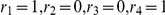). During the reversal phase, the reward function is reversed, meaning that the 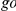 action must be chosen when the stimulus 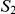 is presented, and vice versa (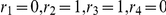). Finally, during the extinction phase, regardless of the presented stimulus, only the 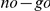 action leads to a reward (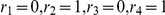). (B) During both the training and reversal phases, subjects' reaction time is high at the early stages when they don't have enough experience with the new conditions yet. However, after some trials, the reaction time declines significantly. Error bars represent 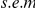.

Using a classical reversal learning task, Pessiglione and colleagues have measured human subjects' reaction time by temporal decoupling of deliberation and execution processes [27]. Reaction time, in their experiment, is defined as the interval between stimulus presentation and the subsequent response initiation. Subjects are required to choose between two alternative responses (“go” and “no-go”), as soon as one of the two stimuli (“
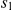
” and “
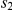
”) appear on the screen. As shown in Figure 6:A, at each trial, one of the two stimuli 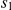 and 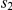 will appear in random, and after the presentation of each stimuli, only one of the two actions results in a gain, whereas the other action results in a loss (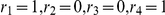). The rule governing the appropriate response must be learned by the subject through trial and error. After several learning trials, the reward function changes without warning (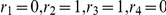). This second phase is called the reversal phase. Finally, during the extinction phase, the “go” action never leads to a gain, and the appropriate action is to always choose the “no-go” response (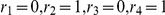).

To analyse the results of the experiments, the authors have divided each phase into two sequential periods: a “searching” period during which the subjects learn the reward function by trial and error, and an “applying” period during which the learned rule is applied. The results show that in the searching period of each phase, the subjects might choose either the right or the wrong choice, whereas during the applying period, they almost always choose the appropriate action. Moreover, as shown in Figure 6:B, the subjects' reaction time is significantly lower during the applying period, compared to the searching period.

**Figure 7:**
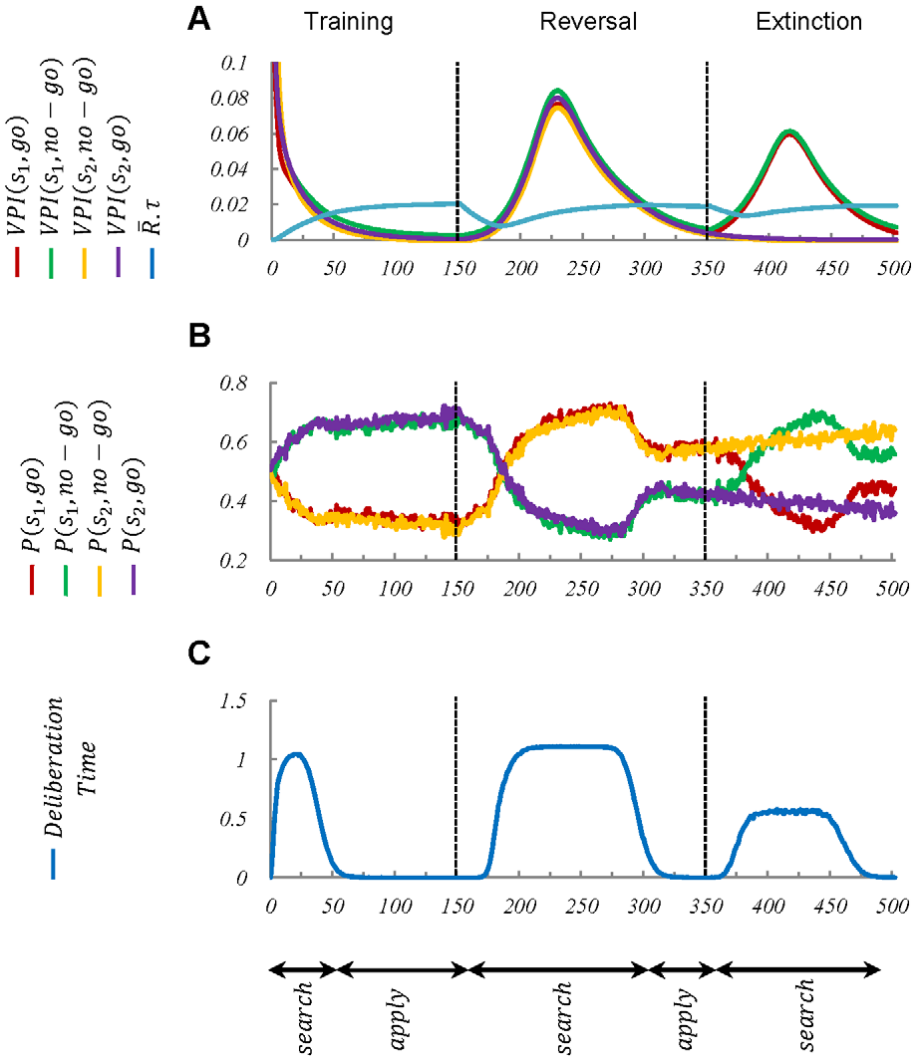
Simulation results of the model in the reversal learning task depicted in **Figure 6**. Since the 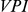 signals have high values at the early stages of learning (plot 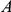), the goal-directed system is active and thus, the deliberation time is relatively high (plot 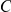). After further training, the habitual system takes control over behaviour (plot 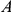) and as a result, the model's reaction time decreases (plot 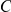). After reversal, it takes some trials for the habitual system to realize that the cached 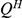-values are not precise anymore (equivalent to an increase in the variance of 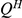). Thus, after some trials after reversal, the 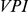 signal increases again (plot 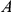), which results in re-activation of the goal-directed system. As a result, the model's reaction time increases again (plot 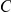). A similar explanation holds for the rest of the trials. In sum, consistent with the experimental data, the reaction time is higher during the searching period, than the applying period.

Figure 7 shows that our model captures the essence of experimental results reported in [27]. In fact, the model predicts that during the searching period, the goal-directed process is involved in decision making, whereas during the applying period, the arbitration mechanism doesn't ask for its help in value estimation. It should be noticed that the reaction time reported in [27], is presumably the sum of stimulus-recognition time, deliberation time, etc. Thus, a fixed value, which is the sum of all the other processes involved in choice selection, must be added to the deliberation time computed by our model.

One might argue that variations in reaction time in the mentioned experiment could also be explained by a single habitual system, by assuming that lack of sufficient learning induces a hesitation-like behaviour. For example, high uncertainty in the habitual system at the early stages of learning a task, or after a change is recognized, can result in a higher-than-normal rate of exploration [18]. Thus, assuming that exploration takes more time than exploitation, reaction time will be higher when the uncertainty of 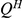-values is high. However, as emphasized by the authors in [27], uncertainty doesn't have any effect on the subject's movement time, but only on the reaction time. In fact, movement time remains constant through the course of the experiment. Movement time is defined as the interval between response initiation and submission of the choice. Since movement time is unaffected by the extent of learning, it is unlikely that variations in reaction time be due to a hesitation-like effect and thus, as an alternative, it can be attributed to involvement of deliberative processes. Moreover, such an explanation lacks a normative rationale for the assumption that exploration takes more time than exploitation.

### Reaction-Time as a Function of the Number of Choices

According to a classical literature in behavioural psychology, choice reaction time (CRT) is fastest when only one possible response is available, and as the number of alternatives increases, so does the response latency. Originally, Hick [28] found that in choice reaction time experiments, CRT increases in proportion to the logarithm of the number of alternatives. Later on, a wealth of evidence validated his finding (e.g., [29]–[35]), such that it became known as “Hick's law”.

Other researchers [36], [37] found that Hick's law holds only for unpracticed subjects, and that training shortens CRT. They also found that in well-trained subjects, there is no difference in CRT as the number of choices varies.

**Figure 8:**
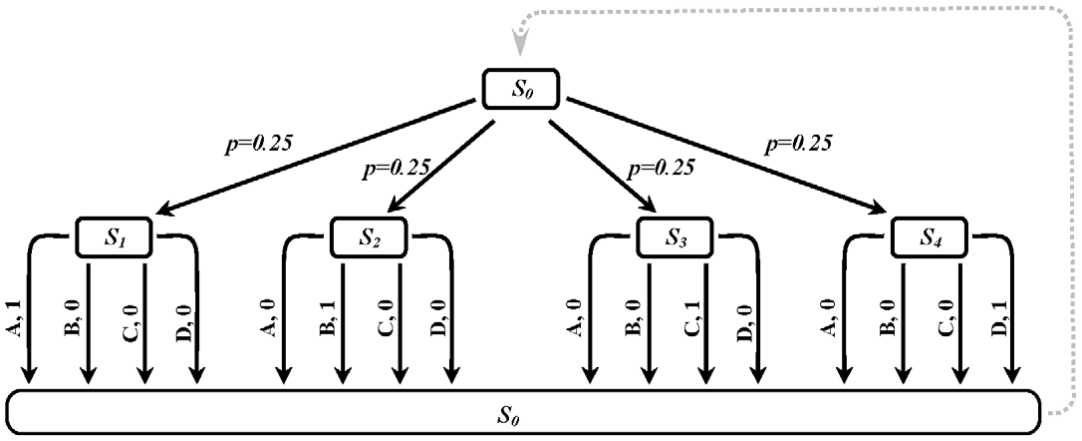
The tree representation of the task for testing the Hick's law. In this example, at each trial, one of the four stimuli is presented with equal probabilities. After observing the stimulus, only one of four available choices lead to a reward (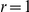). The task structure is verbally instructed to the subjects before they start performing the task. The interval between the appearance of the stimulus and the initiation of a response is measured as “reaction time”. The experiment is performed under different numbers of stimulus-response pairs; e.g. some subjects perform the task when only one stimulus-response pair is available (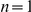), whereas for other subjects the number of stimulus-response pairs might be different.

In a typical CRT experiments, a certain number of stimuli and the same number of responses are used in each session of the experiment. Figure 8 shows the tree representation of an example task with four stimuli and four alternatives. In each trial, one of the four alternatives appears at random, and only one of the four responses results in a reward. As in the CRT experiments the subjects are provided with a prior knowledge about the appropriate response after the presentation of each stimuli, we assume that this declarative knowledge can be fed into and used by the goal-directed system in the form of transition and reward functions. Furthermore, subjects are asked to make true responses, and at the same time as fast as possible. Hence, since subjects know the structure of the task in advance, they show very high performance (as defined by the rate of correct responses) in the task.

**Figure 9:**
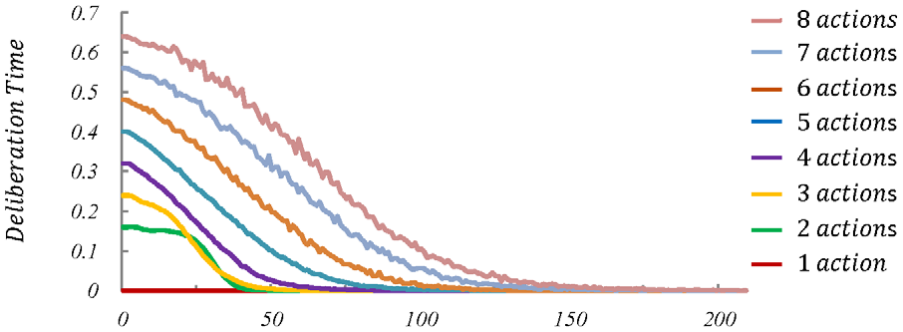
Simulation results for the task of **Figure 8**. Consistent with the behavioural data, the results show that as the number of stimulus-response pairs increase, the reaction time also increases. Moreover, if extensive training is provided to the subjects, the reaction time decreases and becomes independent from the number of choices.

As demonstrated in Figure 9 , the behaviour of the model has replicated the results of CRT experiments: at the early stages of learning, the deliberation time increases as the number of choices increases, whereas after sufficient training, no difference in deliberation time can be seen. It must be mentioned that in contrast to behavioural data, our model predicts a linear correlation between the CRT and the number of alternatives, rather than a logarithmic function. Again, a fixed value characterizing stimulus-identification time must be added to the deliberation time computed by our model in order to reach the reaction time reported in the CRT literature.

Since in CRT experiments a declarative knowledge about appropriate responses is provided to the subjects, they have a relatively high performance from the very beginning of the experiment. The proposed model can explain this behavioural characteristic due to the fact that at the early stages of the experiment, when the habitual system is totally ignorant about the task structure, the goal-directed system controls the behaviour and exploits the prior knowledge fed into it. Thus, a single habitual system cannot explain the performance profile of subjects, even though it might be able to replicate the reaction-time profile. For example, a habitual system that uses a winner-take-all neural mechanism for the 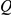-values of different choices to compete [38], [39] also predicts that at the early stages of learning where the 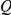-values are close to each other, reaching a state that one action overcomes the others takes longer, compared to the later stages where the best choice has a markedly higher 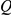-value than other actions. Such a mechanism also predicts that at the early stages, if the number of choices increases, the reaction time will also increase. However, since feeding the subject's declarative knowledge into the habitual system is not consistent with the nature of this system, a single habitual system cannot explain the performance of subjects in Hick's experiment.

## Discussion

### Neural Implications

As mentioned, training-induced neuroplasticity in cortico-basal ganglia circuits is suggested to be mediated by dopamine (DA), a key neuromodulater in the brain reward circuitry. Whereas phasic activity of midbrain DA neurons is hypothesized to carry the prediction error signal [40], [41], and thus imposes an indirect effect on behaviour through its role in learning the value of actions, the tonic activity of DA has shown to have a direct effect on behaviour. For example, DA agonists have been demonstrated to have an invigorating effect on a range of behaviours [42]–[46]. It is also shown that higher levels of intrastriatal DA concentration is correlated with higher rates of responding [47], [48], whereas DA antagonist or DA depletion results in reduced responsivity [49]–[53].

Based on these evidence, it has been suggested in previous RL models that tonic DA might report the average reward signal (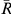) [19]. By adopting the same assumption, our model also provides a normative explanation for those mentioned experimental results, in terms of tonic DA-based variations in deliberation time.

### Rationality of Type II

In the economic literature of decision theory, rational individuals make optimal choices based on their desires and goals [54], without taking into account the time needed to find the optimal action. In contrast, models of bounded rationality are concerned with information and computational limitations imposed on individuals when they are encountered with alternative choices. Normative models of rational choice that take into account the time and effort required for decision making are known as rationality of type II. This notion emphasizes that computing the optimal answer is feasible, but not economical in complex domains.

First introduced by Herbert Simon, it was argued that agents have limited computational power and that they must react within a reasonable amount of time [55], [56]. To capture this concept, [57] used the Scottish word “satisficing” which means satisfying, to refer to a decision making mechanism that searches until an alternative that meets the agent's aspiration level criterion is found. In other words, the search process is continued until a satisfactory solution is found. Borrowed from psychology, aspiration level denotes a solution evaluation criterion that can be either static or context-dependent and acquired by experience. A similar idea has been taken by neuroscientists to explain the speed/accuracy trade-off, using signal detection theory (see [58] for review). In this framework, the accumulated information gathered from a sequence of observations from a noisy evidence must reach a certain threshold, in order for the animals to convert the accumulated information into a categorical choice. If the threshold goes up, the accuracy increases. As in this case more information must be gathered to satisfy that increased level of accuracy, response latency will decrease.

Simon's initial proposal has launched much attempt in both social science and computer science to develop models that sacrifice optimality in favor of fast-responding. The focus has been on complex uncertain environments, where the agent must respond in a limited amount of time. The answer given to this dilemma in social science is often based on a variety of domain-specific heuristic methods [59], [60] in which, rather than employing a general-purpose optimizer, animals use a set of simple and hard-coded rules to make their decisions in each particular situation. In the artificial intelligence literature, on the other hand, the answer is often based on approximate reasoning. In this approach, details of a complex problem are ignored in order to build a simpler representation of the original problem. Finding the optimal solution of this simple problem will be feasible in an admissible amount of time [61].

To capture the concept of time limitation and to incorporate it into models of decision making, we have used the dual-process theory of decision making. The model we have proposed is based on the assumption that the habitual process is fast in responding to environmental stimuli, but is slow in adapting its behavioural strategies, particularly in environments with low stability. The goal-directed system, in contrast, needs time for deliberating the value of different alternatives by tracing down the decision tree, but, is flexible in behavioural adaptation. The rule for arbitrating between these two systems assumes that animals balance decision quality against the computational requirements of decision-making.

However, the optimality of the arbitration rule is based on the strong assumption that the goal-directed decision process has perfectly learned the environmental contingencies. This assumption might be violated at some points, particularly at the very early stages of learning a new task. When both systems are totally ignorant of the task structure, although the habitual system is in desperate need of having perfect information (high 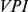 signal), the goal-directed system doesn't have any information to provide. Thus, deliberation not only doesn't improve animal's strategy, but leads to a waste of the time that could be used for blind exploration. Though, since the goal-directed system is very efficient in terms of exploiting the experienced contingencies, this sub-optimal behaviour of the model doesn't last long. More importantly, in real world situations, the goal-directed process seems to always have considerably more accurate information than the habitual system, even in environments that have never been explored before. This is because many environmental contingencies can be discovered by mere visual observation (e.g. searching for food in an open field) or verbal instruction (as in the Hick's task discussed before), without any experience being required.

### State of the Art

Our model is in fact based on the previous computational model of the dual-process theory, proposed by Daw and colleagues [5]. After assigning model-free and model-based RL models to habitual and goal-directed systems, respectively, they suggest an uncertainty-based arbitration mechanism between the two systems. In their model, each of the two systems not only separately estimate a value for each certain action, but their uncertainties about that value-estimations are also computed. As in our model, lack of enough experiences in the environment results in uncertainty in the habitual system. The source of uncertainty in the goal-directed system, on the other hand, is (1) uncertainty in transition and reward functions, due to the lack of enough experiences and (2) “pruning”, which refers to incomplete consideration of the all parts of the decision tree when considering the consequences of alternative choices. The latter source of uncertainty is not explicitly modeled and instead, is captured by adding a noise to the estimated values.

At any given point of time, both systems get involved in value and uncertainty estimation for all the available choices and when they have both finished, the system that is more certain about its estimation of the value of each action will determine the value of that action for action-selection. As a result of this arbitration rule, the goal-directed system is dominant at the early stages of learning; but after extensive learning, the habitual process will take control over behaviour. This happens because uncertainty of the habitual system decreases through the course of learning, whereas the goal-directed process remains uncertain due to the incomplete search of the decision tree (the added noise). Thus, their model can explain the canonical observation in the experimental paradigm of outcome-devaluation (Outcome-sensitivity after moderate, but not extensive training).

The added noise to the goal-directed system in that model actually characterizes, in an adhoc way, all the computational constraints that the goal-directed system is confronted with; e.g. time constraint, working memory constraint, caloric needs, etc. It has also been pointed out in [5], that the trade-off between behavioural flexibility and computational costs can be captured in a cost-benefit fashion. In this respect, the arbitration mechanism we have proposed in this paper is a variant of the model proposed in [5], where only one of the computational constraint, i.e. deliberation time, is modeled in an explicit, cost-benefit account.

Beside this noticeable behavioural harmony of that model with the current dual-process literature, it suffers from some deficiencies. These deficiencies arise from the fact that in that model, the goal-directed system ceaselessly searches for the optimal policy, regardless of the system that is controlling the behaviour. In contrast to this assumption, overtraining of a behaviour is shown to causes a transition in neural activity from the associative to the sensorimotor network; i.e., whereas PFC and caudate nucleus are activated at the early stages of learning a new motor response, this activity shifts to motor cortices and putamen as the response becomes well-trained [62], [63]. As a result, response latency in that model doesn't vary through learning. Of course, it should be mentioned that by adding the noise to the goal-directed system in order to model pruning, time-limitations have been implicitly incorporated into the model; but as this noise level remains fixed through learning, the involvement of the goal-directed system, and so the deliberation time, doesn't change even after extensive training.

As mentioned before, the core idea that we have proposed here for arbitration between the two systems is that there should be a balance between speed and accuracy in responding. A similar idea has been previously used by Shah and Barto [64], but in an evolving sensory representation framework. In the task that they have simulated, subjects must choose among the potential goals in each trial. However, the sensory representation of the true goal of each trial is weak at the beginning of the trial, and resolves gradually during the course of the trial [65]. The basic assumption of their model is that the planning system can select actions only when goal representation is fully resolved, but the habitual system can also use “uncertain” accumulated sensory information. At the early trials of learning the task, since the value of different choices is not learned by the habitual system yet, this system cannot choose among the choices within a considerable period of time. This is due to using a winner-take-all competition mechanism for action selection [38], [39]. Thus, at the early trials, the sensory representation has enough time to be fully resolved and as a consequence of this, the planning system controls behaviour. However, after extended training, the habitual system can make a decision before the goal is fully identified, based on uncertain sensory information.

Although both the model we proposed here and the model proposed in [64] use speed-accuracy trade-off for arbitration between the two systems, there is fundamental differences between them. Whereas the extra time needed by the planning system in is used for state recognition [64], this time is used for deliberating the consequences of choices in our model. In fact, it is the process of state recognition that is time-consuming in their model, and not the process of deliberation. Due to this difference, the model of [64] can only be applied in cases where stimulus identification takes non-negligible time, which doesn't seem to be the case of the experiments addressed by our model.

Changes in the animals' response rate has been previously explained in the reinforcement learning literature [19], [66]. Importantly, in the model proposed by Niv et al. [19], as in our model, animals make a balance between the cost and benefit of acting quickly. 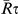 is the cost of responding after an interval 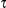. Thus, in their model, as in our model, the animal benefits from responding fast, because it loses less potential rewards. But as they do not model the goal-directed system, the cost of acting quickly in their model is due to an extra fatigue-like cost induced by responding fast, whereas this cost in our model is due to inaccurate and inflexible value estimations. We believe that both factors, influence the animals' response rate.

But as a result of this fundamental difference, the two models have different behavioural predictions. In fact, the term 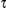 in the model proposed in [19] refers to “execution time”, whereas in our model it refers to “reaction time”. Notice that reaction time is, by definition, the interval between stimulus presentation and performance initiation, whereas execution time (movement time) refers to the interval between response initiation and its finalization. Due to this difference, their model cannot explain any of the three experiments on reaction time that our model can: (1) VTE behaviour, (2) increase in reaction time as the number of choices increases, (3) decrease in reaction time after reversals, in the go/no-go task. Interestingly, by temporal decopulation of deliberation and execution, it has been shown in [27] that whereas reaction time has significantly decreased after reversal in a go/no-go task, the execution time has remained intact.

### Untested Behavioural Predictions of the Model

As mentioned previously, one prediction of the competition mechanism proposed in this paper is that outcome sensitivity is dependent on the relative value of the choices that are concurrently available. That is, if the value of choices are sufficiently close together, the habitual system will remain uncertain about what the best choice is (equivalent to high 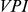), even after extensive training. This will result in the informational gain of knowing the exact value of choices remaining high and thus, the goal-directed system staying dominant. Such a mechanism can explain the behavioural data reported in [26].

By contrast, the model predicts that in a concurrent schedule where the value of the two choices are sufficiently different, responding will eventually become habitual. This is because after extensive training, the habitual system will have sufficient information for choosing the better choice among the two, without needing the exact value of them; i.e., without needing the goal-directed system. To our knowledge, this prediction is not tested yet. In this respect, the model has a different prediction from what the model proposed in [5] predicts. According to that model, the goal-directedness of responding doesn't depend on the relative value of choices and thus, it predicts that responding will remain goal-directed in concurrent schedules, whether the values of choices are equal or not.

Another prediction of our model is that if the two choices in a concurrent schedule lead to a unique outcome, responses will remain sensitive to devaluation, regardless of the amount of instrumental training. This is because when the outcomes are identical, the values of the two choices that lead to it will be exactly the same. In fact, when the values of the two choices are equal, our model predicts that responding will remain goal-directed, whether the identity of the outcomes of choices are the same or not. However, in the model proposed in [5], if the two outcomes are identical, it can be said that since fewer outcome values must be learned, the asymptotic uncertainties of the habitual system will decrease. Thus, according to that model, responding might become habitual or remain goal-directed after extensive training, depending on the parameters of the model.

It should be mentioned that in an experiment by Holland [21], sensitivity to devaluation is tested where two different choices result in an identical outcome. However, since in that experiment responding for the two choices is trained and tested in separate sessions, rather than the choices being available concurrently, the reinforcement learning framework cannot see it as if the values of the choices could be compared together. Therefore, in order to test the above prediction of our model, it is necessary to use a concurrent schedule.

Another theoretical account for competition between the S-R and the A-O systems proposed by Dickinson [67] predicts that competition between the systems depends on the relative value of choices. In this account, responding is goal-directed if, and only if, the animal experiences instrumental contingency between responses and outcomes. Experienced contingency is defined as the correlation between a change in response rate and a change in reward rate. Consistent with behavioural data, this theory predicts that in one-choice tasks where a ratio schedule is used, the response rate and thus the reward rate increase during the initial acquisition period. Hence, due to the positive experienced correlation between the changes in these two variables, responding will be goal-directed. However, after extended training, response rate, as well as reward rate, converge to a high rate. This will remove any experienced contingency perceived by the animal and thus, the habitual system becomes dominant.

For the case of concurrent schedules where the two outcomes are different but have equal values, this account predicts that even after extensive training, the animal might choose either of the two responses from time to time. Thus, every time that the animal performs one of the two responses, it experiences a loss of the outcome that could be acquired by performing the other response. In this respect, the animal always experiences a local correlation between response and outcome rates and thus, remains goal-directed even after extensive training. This prediction is also consistent with behavioural data [26].

However, if the identity of the two outcomes are the same, this theory will have a different prediction. In such a case, since the outcomes are identical, the rate of outcome will be fixed after extensive training regardless of which of the two responses is performed. Thus, in this case, the local A-O rate correlation dies out and responding becomes habitual. Moreover, this account predicts that if the two choices result in different outcomes that have markedly different values, responding will become habitual after extensive training. This is because after extensive training, the high-value choice will become stereotyped and the other response will be chosen rarely. Thus, since only one of the two outcomes is often experienced with a consistently high rate, the locally experienced A-O rate correlation decreases. In fact, the experienced A-O rate correlation is negatively correlated with the difference between the values of the two outcomes: the higher the difference between the values, the lower the experienced instrumental contingency. As a result, if the values of the two outcomes are sufficiently different, responding will become habitual eventually. In this respect, both the theoretical account of [67] and our model predict that arbitration depends on the relative value of the two choices.

A summary of the predictions of the reviewed dual-process accounts are provided in Table 1. The experimental schedules of the first and the third rows of the table, as discussed before, are used in [3] and [26], respectively. As shown, the prediction of all three arbitration mechanisms for these two cases are the same, and supported by behavioural data. However, the theories have differential predictions in the other two cases that are not tested yet.

**Table 1:** Prediction of different dual-process accounts about the dominant process after extensive training.

	Dickinson [67]	Daw et al. [5]	Our model
Single choice	S-R	S-R	S-R
Two concurrent choices with identical outcomes	S-R	S-R or A-O	A-O
Two concurrent choices with different outcomes, but equal values	A-O	A-O	A-O
Two concurrent choices with different outcomes and sufficiently different values	S-R	A-O	S-R

**Figure 10:**
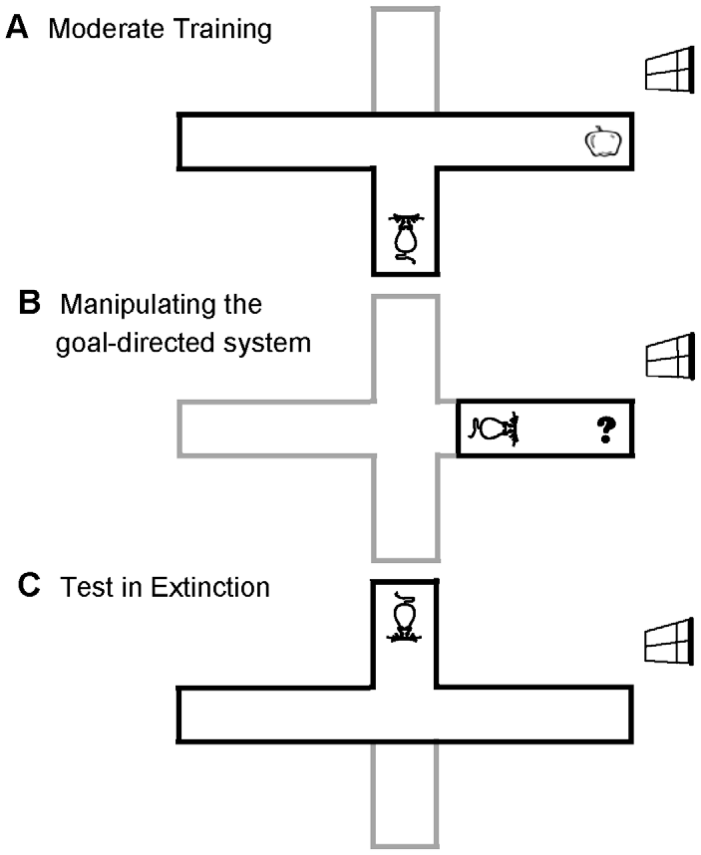
An experiment for testing the validity of the model. The proposed model predicts that manipulating the knowledge acquired by the goal-directed system should not affect the goal-directedness of behaviours. To test this prediction, a place/response task can be used. (A) In the first phase, the animal is moderately trained to acquire food reward in a T-maze. Since this training is moderate, the goal-directed system is expected to control behaviour during this phase. (B) In the second phase, the uncertainty of the goal-directed system is increased by putting the animal inside the right arm for some few trials, while the food reward comes at random or is totally removed. (C) Since the second phase doesn't have any effect on the habitual system, our model predicts that the arbitration between the system must have remained intact and thus, responding should still be goal-directed in the third phase. For that, the animal should still chose turning toward the window, even though its starting point is at the opposite end of the maze.

One critical assumption of our model that is worth being tested is the assumption that arbitration between the systems is independent of any knowledge that is acquired by the goal-directed system. This assumption is in contrast to the model proposed in [5], where the uncertainty of the goal-directed system also plays role in competition among the systems. One way to test this assumption of our model is to manipulate the knowledge of the goal-directed system, while other variables are remained intact, and to test the impact on the goal-directedness of animal's behaviour. For this purpose, a place/response task similar to what is suggested in Figure 10 can be used.

In the first phase, the animal is moderately trained to retrieve food from one arm of a T-maze. Since the training period is moderate, we expect that at the end of this phase, the animal will use a place strategy (goal-directed system) at the choice point, rather than a response strategy (habitual system). Thus, if the animal is then directly tested in the third phase, e.g., the starting arm is placed at the opposite end of the maze, it is expected to still turn toward the window. Now, the critical prediction of our model is that if any manipulation is applied only to the goal-directed system during a new phase between training and test, it should not change the animal's strategy. In fact, our model will be falsified if after such manipulations, the animal chooses the “turn right” response at the choice point (going in the opposite direction of the window), which indicates that it is using the response strategy, rather than the place strategy.

One manipulation is to put the animal inside the right arm for some very few trials, while the food reward comes at random or is totally removed. This will increase the uncertainty of the goal-directed system about the outcome of the strategy “running toward the window”. Note that the number of trials should be sufficiently small such that the animal is not able to learn the new conditions, but only to increase its uncertainty. Among the variables of our model that influence arbitration (i.e., 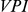, 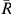, and 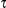), the only variable that is affected due to this manipulation is the average reward variable (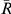). However, since this variable is decreased, the model predicts that such a manipulation will make responding even more goal-directed than before. As the animal has not experienced being at the choice point during the second phase, the habitual system will remain intact in this phase. In sum, our model predicts that whatever the number of trials in the second phase is, the animal must still respond goal-directedly (turn toward the window) in the test phase, even though the second phase has increased the uncertainty of the goal-directed system.

The above experiment is in fact a way to test the hypothesis of the model that outcome-sensitivity after re-exposure (in devaluation experiments) is not the result of shift in control from the habitual to the goal-directed system (through manipulating the goal-directed knowledge during the incentive learning period, as suggested in [5]), but instead, it is because the goal-directed system has been dominant even before devaluation, and the only effect of the re-exposure phase is learning the new incentive value of outcomes (updating the reward function of the goal-directed system). This explanation is the dominant explanation for incentive learning [68]. However, if the rats in the above experiment show response strategy in the third phase (in contrast to what our model predicts), it will support the hypothesis that manipulating the goal-directed system can affect arbitration, and that outcome-sensitivity after devaluation might be due to such a manipulation [5].

Another assumption of our model is that when the animal is at the choice point, the time needed for computing the 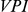, which is in fact the time needed for arbitration, is trivial, compared to the time needed for goal-directed search. As mentioned before, this is a plausible assumption since the 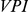 signal can be computed by a closed form equation [18]. However, it might be argued that goal-directed responding can also be achieved within a trivial period of time. This is possible, for example, by assuming that the goal-directed system is capable of evaluating the value of choices in an off-line mode (when the animal is not necessarily performing the task) and caching them for future exploitations. Similarly, the goal-directed system might be argued to be neurally implemented by an attractor equation for value iteration (e.g. [69]). Fortunately, the assumption of our model that goal-directed search requires a considerable time is experimentally testable by measuring the animal's reaction time at the choice points, and comparing them when responding is habitual vs. when it is goal-directed (see Figure 3:I).

### Future Directions

One limitation of the proposed model is that the computation of the average reward signal, which is assumed to be encoded by tonic dopamine, requires the simulated task to be cyclic and highly repetitive. For example, since shifts in the animal's motivational states don't have an immediate impact on the average reward signal, they cannot have a direct effect on the arbitration mechanism. This is despite the fact that motivational states, like hunger and thirst, are demonstrated to modulate the tonic firing activity of dopamine neurons [70], even before new training under the new motivational state being provided to the animal. It is also analytically more reasonable that the opportunity cost be a function of motivational states; e.g. a hungry animal has a higher opportunity cost, compared to a sated one. One way to resolve this limitation is to develop a more realistic formulation for opportunity cost, rather than the simple average reward formulation.

A similar limitation of the model concerns the necessity of experiencing rule changes by the subject, for the arbitration mechanism to be affected. In fact, the model is silent about how an unexperienced, but verbally communicated, environmental change can affect the competition between the two systems. At least in some cases for humans, it seems that a communicated change in the context makes the goal-directed system able to override the habitual response. Modeling such a phenomenon requires a normative way for the arbitration mechanism to be directly influenced by verbal instructions. Although in our model verbal instructions are supposed to affect the subjects' goal-directed knowledge, they don't contribute to the arbitration mechanism.

A critical question that must be answered in any dual-process account of decision making is why animals need two systems. In fact, if the goal-directed system makes more rational decisions, then why the habitual system should have survived? One raw answer to this question could be that animals' brains were not redesigned anew through the course of evolution, but new capabilities were added to the underlying, evolutionarily old brain structures. A more sophisticated answer is that deliberation is subject to some constraints in a way that making habitual responses is more optimal at many choice points. The constraint that our model relies on is the slowness of deliberation. But it can be argued that an increase in response latency is only one of the costs that the animals' decision making machinery must pay for flexibility in sensorimotor coordination; and some other advantages can be counted for the habitual process, each of which is potentially the basis of another normative computational model.

Working memory limitations is another constraint imposed on the goal-directed process. The previously acquired information that the goal-directed system requires for its analysis must first be loaded to working memory. Hence, subject to working memory limitations, the goal-directed system might not be provided with enough materials for an accurate deliberation and so, its response might be less optimal than the corresponding habitual response.

One more comparative advantage of the habitual system is that it seems impossible, or at least very costly to deliberate about more than one issue at a time, whereas the habitual responses involve massively parallel processing [71]. For example, so many habitual responses are made by a taxi driver while he/she is driving, but the deliberative system is involved in only one issue, e.g. finding the shortest path to reach the destination. Another influential factor that seems to favour habitual decisions despite their non-optimality is that goal-directed deliberation consumes more energy than habitual action selection. For example, low availability of blood glucose, which is the main fuel supporting brain function, results in impairments in cognitive tasks [72]. This factor can be captured by adding an energy cost term, 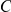 (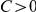), to the cost of deliberation, and hence, for arbitration between the two processes, the 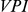 signal must be compared with 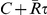.

In both dual-process models proposed in [5] and in this paper, the only type of interaction between the two systems is “competition”. However, collaborative interaction between different associative structures can also facilitate optimal action selection. Among different anatomy-based proposals offered for how segregated cortico-basal ganglia loops might be integrated, the spiral organization of DA neurons have proved compatible with the RL framework. Through these spiral connections between the striatum and the Ventral Tegmental Area/Sabstantia Nigra, the output of more ventral areas of the striatum can affect the functioning of more dorsal regions [73], [74]. Accordingly, it has been hypothesized that by propagating the teaching signal from associative to motor areas of the basal ganglia, more abstract policy representations can facilitate learning habitual motor-level actions [75]–[77]. Based on these evidence, the goal-directed system can be assumed to affect the computation of the prediction error signal, in order to accelerate consolidating the optimal responses in the habitual system. This can substantially resolve the curse of dimensionality in model-free RL, which refers to the exponential growth of learning required for the habitual system when the complexity of the environment increases [78].

### Mathematical Methods

#### Value estimation by the habitual process

The role of the habitual system is to store and update the value of state-action pairs in a cached form, from which high-speed retrieval is possible. If enough experience in provided, the value of each state-action pair, denoted by 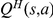, converges to the total discounted rewards expected to be obtained by taking action 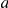 in state 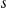 and then following the optimal policy in subsequent states. Regarding that probability distribution functions over 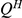-values are required for calculating the 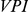 signal, the habitual system also stores and updates an estimation of the accuracy of the learned 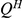-values.

For storing state-action values a look-up table representation is used, which is a special case of the linear parametrization of 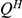-values. For learning 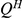-values, we used the 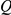-learning version of the Kalman Temporal Differences (KTD) framework proposed in [16]. In addition to learning state-action values, this method provides a measure of accuracy of learned values, which corresponds to the certainty of estimations.

In this framework, the state-space of the problem is formulated as follows:



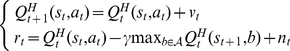
(8)


The first equation implies that 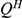-values follow a random walk process. This means that the value of a state-action is composed of its past value plus an evolution noise, 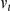 (a Gaussian white noise). The assumption of a process noise for the evolution of 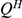-values is necessary because we utilize this framework for the learning of 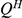-values in a non-stationary MDP, i.e., the reward function of the environment might change over time. The second equation is based on the Bellman equation. 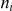 is the observation noise and is supposed to be a Gaussian white noise.

As in the KTD framework where 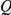-values have distribution functions rather than point estimations, the algorithm keeps track of two matrices: 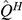, which stores the mean of 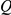-values for different state-action pairs, and 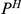, which is the covariance matrix of the former matrix. The diagonal elements of 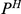 contain the variance of 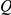-values. The distribution functions over 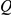-values are assumed to be Gaussian.

Based on this formulation, after taking action 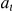 in state 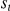 and transiting to a new state, 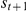, the matrix 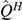 can be updated using the following learning rule:



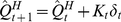
(9)


where 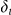 is the temporal difference error, and 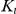 is the Kalman Gain, which determines the direction in which the current representation of values must be corrected. Moreover, after each transition, the covariance matrix is updated using the following equation:



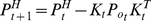
(10)


where 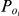 is the estimated variance of the observation equation. The Kalman Gain 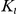 is computed by:



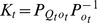
(11)



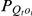
 is the covariance between 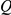-values and the observation equation. Regarding that the observation equation is nonlinear -because of the 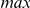 operator-, the values of 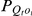 and 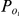 cannot be directly computed from the 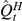 and 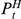 matrices. To address this issue, an unscented transform [79] is used to approximate the statistics of interest [16]. For more details of the KTD algorithm see [16] (Algorithm 5).

Finally, in equation 8 , the covariance matrix of the process noise is chosen in an adaptive way, i.e. 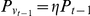.

Since the KTD algorithm used for estimating the mean and the variance of 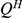-values is computationally expensive (e.g. it involves matrix inversions), one might think that it practically takes the same time that is sometimes withdrawn from goal-directed search. That is, the time necessary for doing the heavy computations of the KTD algorithm must also be taken into account when choosing whether to deliberate or not. However, it must be noticed that at the time that the model is confronted with some choices, all the knowledge required for computing the 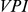 signals (mean and variance of 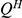-values) is already available in the KTD (habitual) system, without any new computation being required. In fact, all the heavy computations of the KTD algorithm are performed only after a decision is made and the 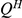 and 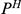 matrices should be updated. Thus, the time required for these computations doesn't influence reaction time.

Moreover, it must be mentioned that the central contribution of the model is in the new arbitration mechanism proposed, and in how the mean and the variance of 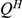-values can be used to make the arbitration rule approximately optimal. In this respect, any algorithm that can give an estimate of the mean and the variance of 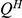-values can be substituted with the KTD algorithm, without affecting the arbitration rule. However, to our knowledge, the KTD algorithm is the most appropriate algorithm, among the currently available algorithms, for the case of the model presented here. The bayesian Q-learning algorithm [18], for instance, updates the 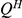-values without using a prediction-error signal and thus, it loses relevance to the dopamine theory.

#### Value estimation by the goal-directed process

Assuming that the goal-directed system has access to an estimation of the reward function, 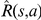, and the transition function, 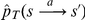, of the environment, then the value of each state-action pair can be calculated using the following recursive equation:
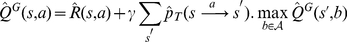
(12)


where 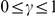 is the discount factor. As the transition graph is cyclic, we impose a maximum limit on the depth of the search. This maximum limit is assumed to be three levels in simulations. After this limit is reached, the recursive process stops and uses the estimated 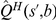 from the habitual system as an estimation of the 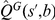 afterward.

The transition function is initialized to 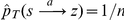, for all 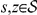 and 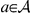, where 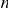 is the total number of states. Assuming that after taking action 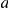 at state 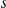, the animal goes to the new state 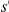, the transition function can be updated using the following rule:
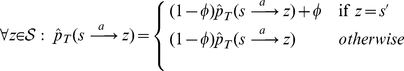
(13)


Where 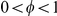 is the update rate of the transition function. This redistribution rule ensures 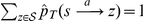 for all 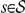 and 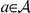.

The estimation of an immediate reward, 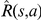, is calculated by taking an exponential moving average over the rewards gained after execution of action 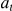 at state 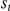 by the agent:
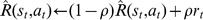
(14)Where 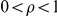 is the update rate of the reward function. For modeling the devaluation of the outcome in the first two simulations, 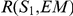 is set to -1.

#### Arbitration between the two processes

When the agent is in state 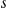, for the purpose of selecting an action among the feasible choices for performance, it needs to have an estimate of the value of each choice. The estimated value of each action can come from either the habitual or the goal-directed process. Thus, for having the final estimated value of each action, the agent has two options: to use values stored in the habitual system or to follow action-outcome contingencies to gain perfect information about state-action values.

If the habitual system is used for acquiring the value of action 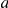 at state 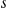, then the animal predicts that it will gain a future reward equal to 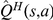, by taking that action. In contrast, if the agent chooses to use the goal-directed system, then the expected sum of discounted rewards will increase by 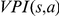 units, due to the policy improvement effect resulted from deliberation. But as it takes 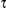 time units for goal-directed value estimation, that extra amount of reward (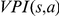) will come after a delay and thus, will be discounted. In fact, by using the goal-directed system, the agent predicts to gain a future reward equal to 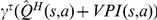, where 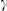 is the discount factor. To act optimally, the agent chooses to deliberate only if it predicts that deliberation will bring it more rewards in future, i.e. 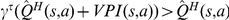. This argument leads to the following decision rule:



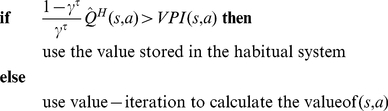
(15)


We are interested in finding a more intuitive equivalent for 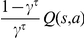. To do so, as proposed in [80], equation 1 can be rewritten as follows:
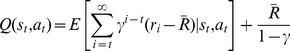
(16)where 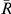 is the average reward calculated over non-exploratory actions, which means that 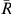 is updated by 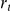, only if the action with the highest expected value has been executed.

In equation 16 , as 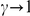, the first term of the above equation tends to the average adjusted value of the state-action pair, which remains finite under some conditions that hold when linear parametrization of values is used and the environment is cyclic [81]. Hence, we will have:



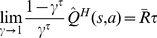
(17)


Using the above equation and assuming that the discount factor has a value close to one, the decision rule noted in equation 15 , can be rewritten as follows:



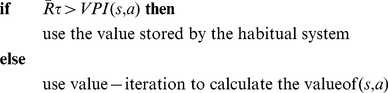
(18)


It is straightforward to show that if rather than the sum of discounted rewards, the goal of the agent was to maximize the average reward signal during its life, then equation 18 would still be an optimal decision rule. 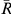 is computed according to equation 7 over non-exploratory actions. For calculation of 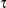, we assume that the time spent for one value-iteration is proportional to the number of edges of the graph traversed during the value iteration process. Also, the time needed to traverse an edge of the graph is assumed to be 0.08 of a time-step. Under these assumptions, we compute the agent's expectation of 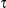 by averaging over the amount of time spent on previous deliberations.

Based on the above discussion, we can define 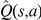, the final estimated value assigned to 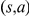 for the purpose of action selection, as follows:
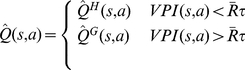
(19)


As illustrated, this value has come from the habitual or the goal-directed process, depending on the result of arbitration. According to this valuation, action selection will be carried out using the softmax action selection rule:
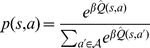
(20)where 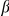 is inverse temperature and determines the rate of exploration.

Finally, assuming that each state-action value has a normal distribution as 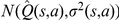, then based on equation 6, 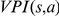 can be calculated as follows [18]:



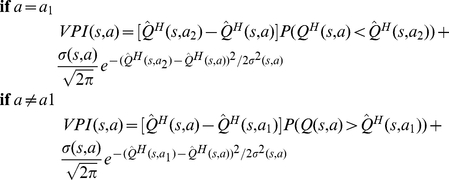
(21)


where 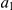 and 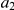 are the best and the second best actions at state 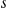, respectively.

**Simulation details.** Table 2 shows the free parameters of the model and their assigned values in simulations.

**Table 2:** Free parameters of the model and their assigned values.

Value	Range	Symbol	Free Parameter
0.02	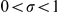	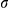	Updating Rate of the Average Reward
0.0001	-	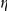	Used to Determine Process Noise
0.05	-	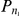	Variance of Observation Noise
1	-	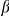	Rate of Exploration
0.1	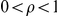	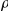	Update Rate of the Reward Function
0.95	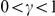	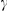	Discount Factor

We showed before that one requirement for the proposed switching mechanism between the two systems to be statistically optimal is that the discount factor, 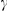, should be sufficiently close to one. However, as the MDPs of the simulated tasks are cyclic, setting 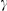 equal to one is nonsense (it will result in non-converging, infinitely large 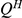-values). Thus, in simulations, 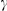 is set very close to one (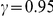).

Since 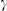 is close to one, 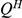-variables converge to relatively high values. However, as 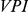 is only affected by the relative value of 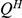-variables, and not their absolute values, the parameter 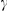 does not affect 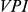 and thus, does not affect the temporal dynamics of arbitration directly.

On the other hand, since a softmax action selection rule is used, the absolute value of 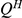-variables also becomes important. In fact, high values of 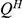-variables caused by the high value of 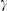 decreases the probability of better actions to be chosen at the action selection phase. This is why the model has chosen at best 60% in Figure 3:H, although the difference between the 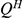-values of the two actions is remarkable (Figure 3:J). Of course, this effect can be easily controlled by adjusting the exploration rate, 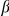. Higher values of 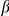 will result in relatively higher probability of selecting the best action.

In sum, although the value of 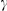 does not affect the arbitration mechanism directly, since it changes action selection probabilities, it influences the convergence speed of 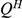-values and thus, affect the arbitration mechanism indirectly. However, it is shown through some simulations that different values of 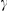 and 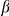 do not change the essence of the behaviour of the model, but only affect the exact time at which switching from one system to the other happens.
